# Apatinib treatment is effective for metastatic malignant phyllodes tumors of the breast: a case report

**DOI:** 10.1186/s12905-021-01359-5

**Published:** 2021-05-22

**Authors:** Xiaolu Wang, Li Xie, Wenjing Hu, Jing Yan, Xiaoping Qian, Lijing Zhu

**Affiliations:** The Comprehensive Cancer Centre of Drum Tower Hospital, Medical School of Nanjing University & Clinical Cancer Institute of Nanjing University, Nanjing, China

**Keywords:** Apatinib, Malignant phyllodes tumors (MPT), Treatment, Case report

## Abstract

**Background:**

We report a rare case of malignant phyllodes tumors (MPT) with partial response to apatinib.

**Case presentation:**

A 26-year-old woman had a palpable mass in her right breast for over a year. After resection, pathology indicated malignant phyllodes tumor. Eleven months after surgery, she underwent reoperation for a lung nodule, which demonstrated lung metastasis. She refused chemotherapy and was rehospitalized six months later due to leg pain. Pelvic mass biopsy revealed metastatic malignant phyllodes tumor. After concurrent chemoradiotherapy of the pelvic mass, multiple lung metastases emerged. Subsequent treatment with apatinib 500 mg/day resulted in a reduction in mass size and partial response. She survived for more than 8 months.

**Conclusion:**

The present case showed the potential therapeutic effects of apatinib in patients with MPT.

## Background

Phyllodes tumors are rare fibroepithelial lesions and account for 0.3–1% of breast cancers [1]. The World Health Organization (WHO) classifies phyllodes tumors as benign, borderline, or malignant [2]. Malignant phyllodes tumors (MPT) have a poor prognosis, with most patients dying within 3 years after starting treatments [1]. Lumpectomy and mastectomy with negative margins remain the preferred treatment options for all types of phyllodes tumors [3]. Radiotherapy is applied for specific situations [4]. There is little evidence of the efficacy of chemotherapy or hormonal therapy, even with estrogen receptor (ER) or progesterone receptor (PR) positivity, in treating MPT. Evidence for favorable effects of systemic therapy for metastatic disease on overall survival (OS) is lacking. Apatinib (Hengrui Pharmaceutical Co., Ltd., Shanghai, China) is a small-molecule tyrosine kinase inhibitor targeting vascular endothelial growth factor receptor 2 (VEGFR-2) and has been proven to be effective and safe for multiple solid tumors [5]. However, there have been no reports of apatinib in the treatment of MPT. In this study, we present a case of metastatic MPT of the breast that was effectively treated with apatinib.

## Case presentation

**Fig. 1:**
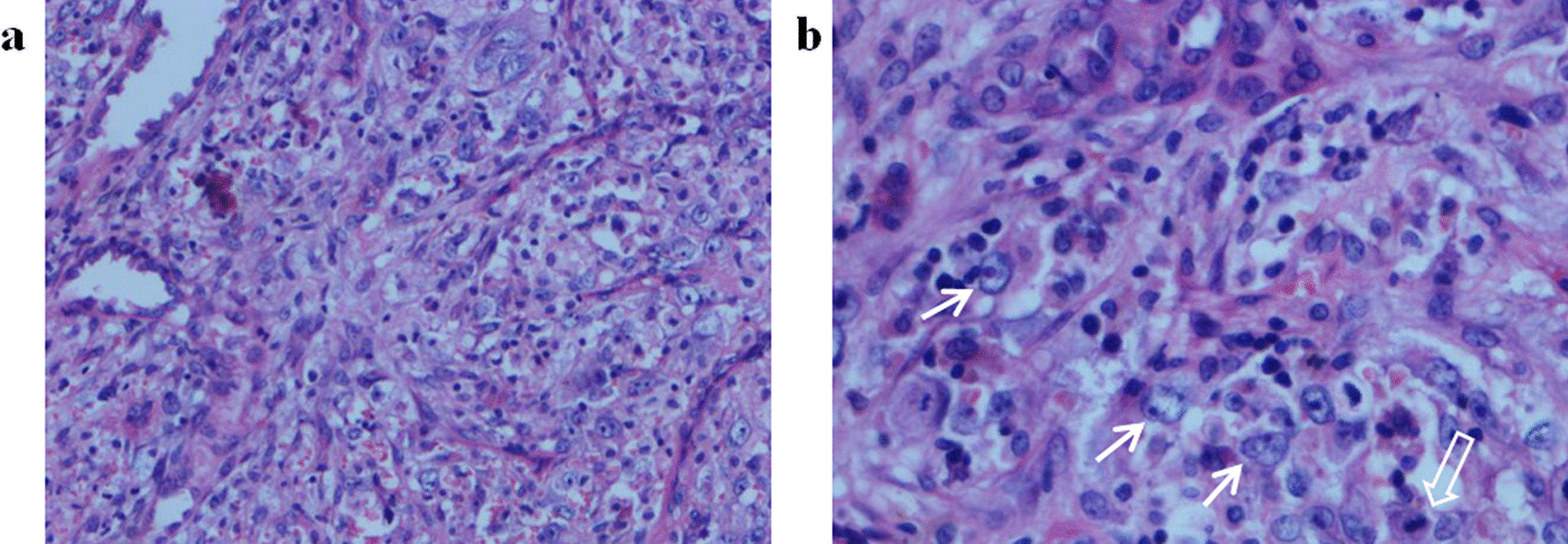
Hematoxylin and eosin (H&E) staining of a tumor section in the left lung. **a** 200 × magnification. The tumor biopsy shows marked stromal cellularity with high pleomorphism. **b** 400 × magnification. The tumor cells are spindled shaped with markedly increased cellularity (white arrows) and a high mitotic rate (hollow arrow)

On November 7, 2016, a 26-year-old Chinese woman was admitted to Drum Tower Hospital because of a palpable mass in the right breast that had been present for over a year. The patient underwent a right mastectomy and sentinel lymph node biopsy. Pathological and histopathological examination revealed a breast MPT measuring 17 cm in in maximum diameter. The lateral and basal margins were clear. Immunohistochemistry (IHC) showed Bcl2 ( +), P53 (+ + +), CD34 (Vessel +), Desmin (−), SMA (−), S100 (−), ER (−), PR ( ±), and HER2 (−). The Ki-67 proliferation index was 70%. On June 2, 2017, follow-up computed tomography (CT) showed a lesion in the left lung. The lesion was then resected by thoracoscopy. Pathological examination indicated metastatic MPT (Fig. 1). The size of the lesion was approximately 2 cm in maximum diameter, and IHC revealed ER (−), PR (−), and HER2 (−) with a high Ki-67 index (approximately 50%). No mutation of *C-KIT* or *PDGFRA* was detected by gene sequencing. Chemotherapy was recommended, but the patient refused it.

Six months later, the patient returned with persistent pain in the left hip. Positron emission tomography-CT showed a pelvic mass with increasing 18F-FDG uptake. A percutaneous pelvic mass biopsy was performed, revealing a MPT metastasis. IHC showed PR (+), P16 (+ + +), ER (−), CD34 (−), CD117 (−), S100 (−), desmin (−), MA (−), and HER2 (−), and the Ki-67 index was 60%. Real-time RT-PCR analysis of the tumor specimens indicated that *BRCA1* was moderately expressed and *RRM1* was highly expressed and that the expression of *TOP1* was somewhere between moderate and high. Palliative radiotherapy for left pelvic metastatic lesions was conducted from January 16 to February 11, 2018. A total dose of planning target volume(PTV) 50 Gy/10 fractions and planning gross tumor volume (PGTV) 70 Gy/10 fractions was prescribed concurrently with paclitaxel liposome 120 mg per week. The reset CT scan on January 31, 2018, showed that the real component of the mass decreased. The pain was significantly relieved from 6 to 1 on the pain scale, and the patient was able to walk freely after radiotherapy.

**Fig. 2:**
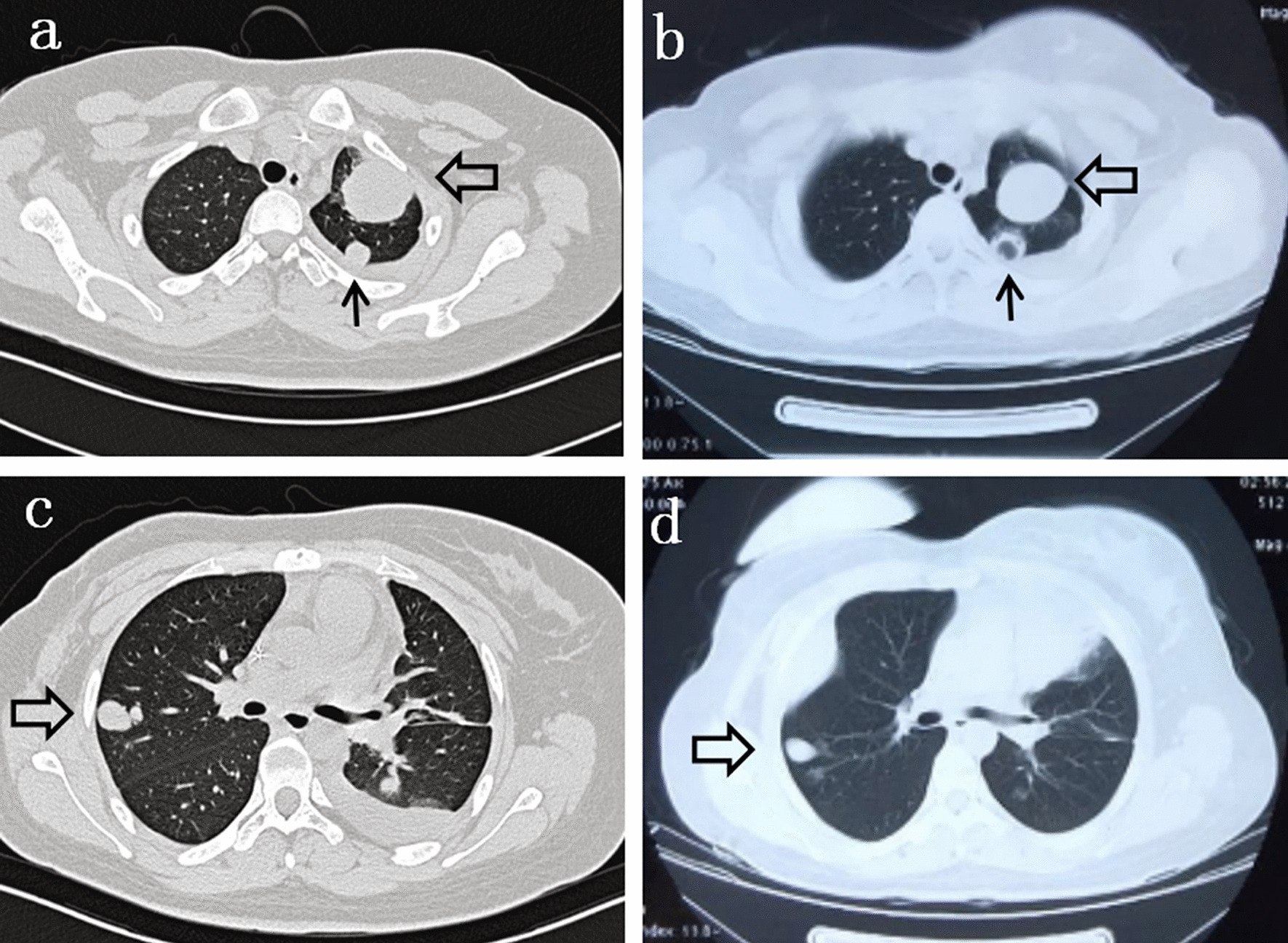
Chest CT scans before and after apatinib therapy. **a** Before apatinib therapy (March 09, 2018), a CT scan showed solid nodules. **b** After 2 months of apatinib treatment (May 11, 2018), one of the solid nodules was reduced (hollow arrows) and the other one transformed to a cystic nodule (black arrows). **c** Before apatinib therapy (March 09, 2018), a CT scan showed the mass (hollow arrow). **d** After 2 months of apatinib treatment (May 11, 2018), the mass was smaller than 2 months before (reduced in size by 15%)

After approximately one month, the patient returned to the hospital with cough, chest tightness, wheezing, and slight hemoptysis. The CT scan revealed multiple lesions in both lungs (Fig. 2), while the pelvic mass remained stable. On March 12, 2018, apatinib was given after the patient provided written informed consent, at a daily dose of 500 mg. After two weeks, symptoms were observably improved. Two months later, the chest CT scan at the local hospital showed a significant reduction in lesions, some of which were vacuolated (Fig. 2). According to the Response Evaluation Criteria in Solid Tumors (RECIST) (version 1.1), target lesions are defined at baseline and must be ≥ 10 mm in longest diameter or ≥ 15 mm in short axis if the lesion is a lymph node. A maximum of 5 lesions may be chosen, with a maximum of 2 per organ. The sum of all the extra-nodal long axis measurements and nodal short axis measurements is calculated. A decrease in the sum of target disease of ≥ 30% represents partial response. In this case, partial response was achieved according to the RECIST guideline (version 1.1) [6]. The adverse effects, transient and slight hemoptysis, could be relieved by oral hemostatic treatment. Apatinib therapy was continued for more than 8 months, and the patient was in good condition. At the last follow-up visit on December 1, 2018, the patient was still alive.

## Discussion and conclusion

MPTs constitute 20% of all phyllodes tumors, are highly aggressive and have a tendency to form distal metastases [7]. The average recurrence free survival is 20.2 ± 12.1 for benign tumors, 16.9 ± 10.8 for borderline tumors, and 20.3 ± 19.0 months for MPT [8]. When a diagnosis of phyllodes tumors is suspected, extensive imaging and histopathological examinations are essential [9]. A core biopsy is recommended if the mass has a smooth outline, intramural contours, and low echogenicity on ultrasound [10]. Microscopically, phyllodes tumors lack real capsules and have increased mild stromal cellularity, mild nuclear atypia, and leafy structures.

Currently, the treatment for MPT remains challenging, with no standard therapeutic regimen. According to the National Comprehensive Cancer Network (NCCN) guidelines, the recommendation for treatment of MPT is complete surgical resection with at least 1-cm margins without sentinel lymph node biopsy. The usage of radiotherapy has also recently increased. Nevertheless, the reduced local recurrence but lack of disease-free or OS advantage has been a limiting factor in conventional radiotherapy [11, 12]. In the present case, palliative radiotherapy for metastatic lesions was intended to alleviate the symptoms. The use of chemotherapy is controversial due to the poor prognosis and frequency of recurrence in metastatic disease. The positive rates of ER and PR are 58% and 75% for phyllodes tumors, but no significant effect has been observed with hormone therapy [13].

Many pathways and molecules are related to the pathogenesis of phyllodes tumors. The expression of several biomarkers, including P53, C-KIT, CD10, and epidermal growth factor receptor, has been investigated [14]. Nevertheless, these biomarkers have limited clinical value in predicting tumor behavior and classification. Vascular endothelial growth factor (VEGF) is involved in angiogenesis and endothelial cells. As phyllodes tumors progress, overgrowth of the stroma may lead to relatively hypoxic areas, triggering the expression of hypoxia-inducible factor-1a (HIF) and VEGF, thereby promoting angiogenesis and increased microvessel density [15]. Although stromal expression of VEGF in phyllodes tumors is reported to increase significantly with increasing grade, data on VEGF expression in phyllodes tumors are still limited [16]. Ho et al. performed immunohistochemistry on tissue microarrays of phyllodes tumors, and the results showed that VEGF in stromal cells was expressed in 64.7% of MPTs. They also found that patients whose tumors expressed VEGF had poorer OS [17]. Therefore, targeting VEGF may be a good treatment strategy for phyllodes tumors.

Tumor angiogenesis plays a vital role in the initiation, progression and metastasis of tumors. Antiangiogenic therapies is a promising anticancer treatment strategy. Tyrosine kinase inhibitor (TKI) targeting VEGFR has led to remarkable advances in the treatment of many tumors. At least 20 VEGFR-TKIs (sorafenib, sunitinib, pazopanib, regorafenib, imatinib, etc.) have been approved by the FDA, all TKIs have shown promising preclinical and clinical effectiveness against sarcoma. However, it is reported that the efficacy of apatinib is comparable or even superior to other TKIs [18]. Its antitumor characteristics include vascular normalization, tumor regression, tumor microenvironment optimization. Nevertheless, due to the low incidence of MPT, there have been no studies of antiangiogenic therapies in MPT. Considering the similarities between MPT and sarcoma, we can refer to researches on sarcoma for clinical strategies.

Apatinib is a TKI that targets the VEGFR2 signal and exhibits a powerful antitumor effect in a variety of solid tumors [5, 19]. It was launched in China in 2014 and has been proven to be effective in advanced gastric cancer, metastatic breast cancer, esophageal cancer and non-small cell lung cancer^5^. Nevertheless, it is unclear whether apatinib affects the treatment of phyllodes tumors. Sarcoma, a group of heterogeneous malignant tumors derived from mesenchymal tissue, is an indication of VEGF/VEGFR targeted therapy [20]. Thus, based on the similarity of phyllodes tumors to sarcomas and the relative overexpression of VEGF, we speculated that apatinib may be effective in phyllodes tumors. The maximum tolerated dose of apatinib is 850 mg once per day. Regarding the safety of apatinib, the most common drug-related adverse events were hypertension (69.5%), proteinuria (47.8%) and hand-foot syndrome (45.6%) [21]. Two retrospective studies of apatinib in the treatment of sarcoma have been conducted, both of which reported no drug-related severe adverse effects (AEs) [22, 23].

In this case, the patient had undergone surgeries at the primary site and for lung metastasis; however the disease continued to progress, and her physical condition deteriorated accordingly. The gene analysis showed no mutation in *C-KIT* or *PDGFRA*, excluding the indication for imatinib, a *C-KIT* and *PDGFRA* inhibitor. Then, we performed real-time RT-PCR analysis, and the results of mRNA expression testing of *BRCA1*, *TOP1*, and *RRM1* implied that the patient might be sensitive to paclitaxel, irinotecan, and gemcitabine. Then, we chose paclitaxel as a radiosensitizer for radiotherapy. The concurrent chemoradiotherapy effectively relieved the symptoms. However, the lesions in the lungs progressed. For systemic treatment, we referred to the treatment of sarcoma and used apatinib. Apatinib successfully reduced the mass size in the lungs. The survival time had exceeded 8 months since the treatment with apatinib, but she has since been lost to follow-up. There was no serious toxicity except for controllable and well-tolerated hemoptysis. Thus, the long-term efficacy of apatinib for phyllodes tumors can be expected.

The present case showed the potential therapeutic effects of apatinib in patients with MPT. To our knowledge, this is the first report of apatinib in the treatment of phyllodes tumors. For advanced cases with multiple metastases, finding an effective targeted drug is very important for patient prognosis and quality of life. Further cohort and prospective trials are needed to identify a subset of patients suitable for apatinib in the clinical treatment of phyllodes tumors.

## Data Availability

Not applicable.

## Abbreviations

MPT: Malignant phyllodes tumors
WHO: World Health Organization
ER: Estrogen receptor
PR: Progesterone receptor
OS: Overall survival
VEGFR-2: Vascular endothelial growth factor receptor 2 CT computed tomography
RECIST: Response Evaluation Criteria in Solid Tumors
PTV: Planning target volume
PGTV: Planning gross tumor volume
NCCN: National Comprehensive Cancer Network
VEGF: Vascular endothelial growth factor
HIF: Hypoxia-inducible factor-1a
TKI: Tyrosine kinase inhibitor
AEs: Adverse effects
